# Previous obstetrical history does not impact short-term mid-urethral sling outcomes

**DOI:** 10.1007/s00192-021-04836-5

**Published:** 2021-05-14

**Authors:** Ole Aleksander Dyrkorn, Anne Cathrine Staff, Sigurd Kulseng-Hanssen, Rune Svenningsen

**Affiliations:** 1 Division of Obstetrics and Gynaecology, Oslo University Hospital, Ullevål, PB 4950, Nydalen, 0424 Oslo, Norway; 2 Faculty of Medicine, University of Oslo, Oslo, Norway; 3 The Norwegian Female Incontinence Registry, Oslo University Hospital, Oslo, Norway

**Keywords:** Stress urinary incontinence, Mid-urethral sling, Pregnancy, Obstetric delivery

## Abstract

**Abstract:**

**Introduction and hypothesis:**

Pregnancy and delivery are known risk factors for stress and mixed urinary incontinence. The most common surgical treatment is mid-urethral sling (MUS) surgery. This study evaluated the potential impact of the obstetrical history on the short-term subjective and objective failure rates after MUS surgery.

**Methods:**

A registry-based surgical cohort study using data from the Medical Birth Registry of Norway (MBRN) and the national Norwegian Female Incontinence Registry (NFIR). Data from 14,787 women that underwent MUS surgery from 1998 to 2016 with complete registrations in the MBRN were included. Outcomes were 6–12-month postoperative subjective and objective failure rates. The potential impact of obstetrical and constitutional factors on both outcomes was tested in a multivariate logistic regression model.

**Results:**

Several obstetrical variables seemed to impact both outcomes in the univariate analyses. However, in the multivariate analyses, none of the obstetrical variables significantly impacted subjective failure, and only being nulliparous before MUS surgery remained a risk factor for objective failure [aOR 1.60, (95% CI 1.07–2.40), *p* = 0.022]. High body mass index at time of surgery, non-retropubic slings, high preoperative urgency symptom load, and surgical complications were all strong risk factors for poor outcomes in the multivariate analyses.

**Conclusion:**

Although childbirth is considered a risk factor for developing stress urinary incontinence, childbirth does not appear to affect the result of MUS in parous women. Our results suggest that nulliparous women with SUI may have a different pathophysiology than SUI after childbirth.

**Supplementary Information:**

The online version contains supplementary material available at 10.1007/s00192-021-04836-5.

## Introduction

Female urinary incontinence (UI) is the most prevalent long-term pelvic floor disorder with a substantial negative impact on women’s quality of life, productivity, socializing, and sexuality [1, 2]. Stress urinary incontinence (SUI), defined as involuntary loss of urine on physical exertion, sneezing, or coughing [3], affects approximately 15% of the female population, with the highest prevalence found in women between 25 and 49 years of age [1].

Although the etiology of SUI is multifactorial, childbirth is uniformly considered a significant risk factor [4–7]. The underlying pathophysiology has not been entirely determined, but it has been proposed that pelvic floor injuries may weaken the supportive structures around the bladder neck and urethra through mechanical forces such as compression, stretching, or tearing of nerves, muscles, and/or connective tissue, causing SUI [8]. However, there is an ongoing debate regarding the possible long-term effects of pregnancy itself on later pelvic floor dysfunction and the additive effects of vaginal delivery in combination with other risk factors, such as birth weight, operative vaginal delivery and maternal age. There is also some evidence of a protective effect on pelvic floor function from cesarean delivery in the short term compared to vaginal delivery, but long-term protection has been questioned [4, 5, 9].

Surgical treatment is usually recommended when conservative treatment options fail [10]. Over the last decades, mid-urethral slings (MUS) have been established as the standard surgical treatment for SUI and stress-dominant mixed urinary incontinence (MUI) mainly due to their high efficacy, long-term durability, and lower rates of repeat surgery compared to other incontinence procedures [11]. Despite the high cure rates, there is still a group of women for whom the surgery fails in obtaining continence. Some of the risk factors identified are age at time of surgery, obesity, concomitant pelvic organ prolapse surgery, and surgical technique [12–16].

Even though childbirth is considered a significant risk factor for SUI development, we have to date little knowledge on whether the same obstetrical factors that caused injuries to the pelvic floor leading to SUI could potentially also determine the success of later SUI surgery. Norway has maintained national medical registries for many years. A national compulsory birth registry has been in place since 1967 and a national quality registry for female urinary incontinence surgery since 1998. Combining these two registries, we aimed to investigate the potential impact of various obstetrical factors on short-term subjective and objective failure rates after MUS surgery.

## Materials and methods

This was a registry-based surgical cohort study of women recorded in the national Norwegian Female Incontinence Registry (NFIR) as having undergone MUS surgery for either stress or stress-dominant mixed urinary incontinence in Norway from 1998 to 2016. As the main aim of the study was to evaluate the impact from previous obstetrical history on surgical outcomes of later MUS operations, data on these women from the NFIR were then merged with data from the Medical Birth Registry of Norway (MBR) from its inception to 2016.

The Medical Birth Registry of Norway (MBRN) was established in 1967 as a mandatory population-based registry for all deliveries in Norway with nearly complete coverage. It receives notification of all deliveries in Norway with information on maternal health as well as prenatal, obstetrical, and neonatal outcomes.

The NFIR was established in 1998 and is a national quality registry for women undergoing urinary incontinence surgeries in Norway [17]. Participating is not mandatory, but > 95% of all female incontinence operations in Norway are performed in public hospitals that regularly report to the registry. The registry is used to improve the quality of incontinence surgeries at reporting hospitals by comparing results and complication rates to a national average in addition to collecting data for research. In recent years the registry has achieved almost complete coverage of women undergoing incontinence surgery and shown high reliability of reported and stored data [18]. Hospitals performing female incontinence surgery report their preoperative and 6–12-month postoperative subjective and objective data to NFIR. Subjective data are collected using a validated short-form urinary incontinence disease-specific questionnaire that calculates indices for the load of stress and urgency symptom bother [19]. The stress and urgency urinary incontinence indices are calculated from two domains of clustered questions and range from 0 to 12 (stress) and 0 to 8 (urgency), respectively. A high index score indicates a high symptom load with 0 indicating no symptoms. In addition, objective clinical data are reported by the surgeon at the time of the operation including results from preoperative objective testing, urodynamic findings, type of incontinence procedure, and any surgical complications occurring at or following surgery. Lastly, subjective and objective data from a mandatory follow-up 6–12 months after surgery are reported using the same validated questionnaire for subjective data as well as reporting results from objective testing, uroflowmetry, and post-void residual urine measurements. At the time of data extraction, 29 of the 39 hospital departments performing female incontinence surgery in Norway reported to the registry, which included approximately 70% of the incontinence surgeries performed in Norway during this time period (data given to authors from the NFIR).

The two outcomes for this study were short-term (6–12-month) subjective and objective SUI failure rates after primary MUS surgery. Patients with concomitant pelvic organ prolapse (POP) surgery or lacking information on whether the surgery was a primary or recurrent SUI operation were excluded. Subjective failure was defined as a stress urinary incontinence index score > 0 at the postoperative 6–12-month follow-up. Objective failure was defined as an increase in pad weight ≥ 1 g during a standardized cough-jump pad-weighing stress test consisting of 20 jumping jacks on the spot and three forceful coughs in the standing position with 300 ml bladder volume [20]. Women who were registered in the NFIR as having undergone a later operation for recurrent SUI after previous MUS before the 6–12-month follow-up were defined as both subjective and objective failures in the statistical analyses.

From the NFIR, the following data were extracted: date of surgery, type of mid-urethral sling (retropubic, inside-out obturator, outside-in obturator, or mini-sling), body mass index (BMI) at time of surgery, preoperative post-void residual urine, preoperative maximum urinary flow rate, maximum urethra closure pressure, pre- and 6–12-month postoperative results from the cough-jump pad-weighing stress tests, pre- and 6–12-month postoperative stress, and urgency urinary incontinence indices and complications (yes/no) during or immediately after surgery (complete list of complications provided in supplemental Appendix 1).

From the MBRN, the following data were extracted: parity, mode of delivery, offspring birth weight, head circumference, presentation, episiotomy, grade 3 and 4 perineal tears, and mothers’ age at delivery. In addition, time from last delivery until MUS surgery was calculated. To ensure complete data from the birth registry and accurately determine which women were nulliparous at the time of surgery, we chose to exclude women born before 1949 as these could theoretically have given birth to non-registered children before the inception of the MBRN.

Potential obstetrical and constitutional risk factors were tested against both subjective and objective failure as previously defined, using a multivariate logistic regression model. The risk factors evaluated in the model were parity (0, 1, 2, or ≥ 3), mode of delivery, presentation, episiotomy (yes/no), offspring birth weight (< or ≥ 4000 g), head circumference (< or ≥ 36 cm), perineal tear grade 3 or 4 (yes/no), age at first delivery (< or ≥ 25 years), age at surgery (in decades), BMI at surgery (normal weight: 18.5–24.9, overweight: 25.0–29.9, and obese: ≥ 30.0 as defined by the World Health Organization), time from last delivery until surgery (in decades), preoperative stress and urgency urinary incontinence index scores (in quartiles), preoperative cough-jump pad-weighing stress test results (in quartiles), preoperative post-void residual urine (< or ≥ 100 ml), preoperative maximum urinary flow rate (< or ≥ 15 ml/s), type of mid-urethral sling (4 groups), and surgical complications (yes/no). Fetal macrosomia, defined as ≥ 4000 g, was used as a dichotomization of birth weight. Head circumference and age at first delivery were dichotomized around the median. Mode of delivery was stratified in three groups (spontaneous vaginal only, instrumental vaginal, cesarean only). Women who had at least one instrumental vaginal delivery were stratified to the instrumental vaginal delivery group regardless of whether they had also had cesarean or spontaneous vaginal deliveries. Women stratified to the cesarean group had only had cesarean deliveries.

Methods, definitions, and units in the study conform to the standards recommended by the International Urogynecological Association and International Continence Society joint report on the terminology for female pelvic floor dysfunction [3].

The study was approved by the Regional Committee for Medical and Health Research Ethics in Norway (#2015/434) and the Institutional Personal Data Officer at Oslo University Hospital. Exemption from the requirement for patient consent was given because there was no foreseen risk of violation of personal data in the study combined with the need for completeness of data for study validity. Full anonymity of participating women was maintained in the final study file used for data analysis. The study was registered at ClinicalTrials.gov (NCT02999347).

Statistical analyses were performed using STATA: Software for Statistics and Data Science version 16.0 (StataCorp). Continuous variables are presented as medians with interquartile range (IQR), and dichotomous variables are presented as frequencies (percentages). Mann-Whitney U-test was used to compare continuous variables and Fisher's exact test for dichotomous variables. Potential risk factors for objective or subjective short-term failure after MUS are presented as crude and adjusted odds ratios (OR) with 95% confidence intervals (CI) and *p*-values in the logistic regression model. A significance level of 5% was used. Covariates with a significance level of 0.20 in the univariate crude analysis were included in the multivariate regression model. A backward variable selection was then used by stepwise removal of variables with significance levels > 0.05. No significant interactions were found between the independent variables. The final model was tested using a goodness-of-fit test (Hosmer-Lemeshow).

## Results

**Fig. 1:**
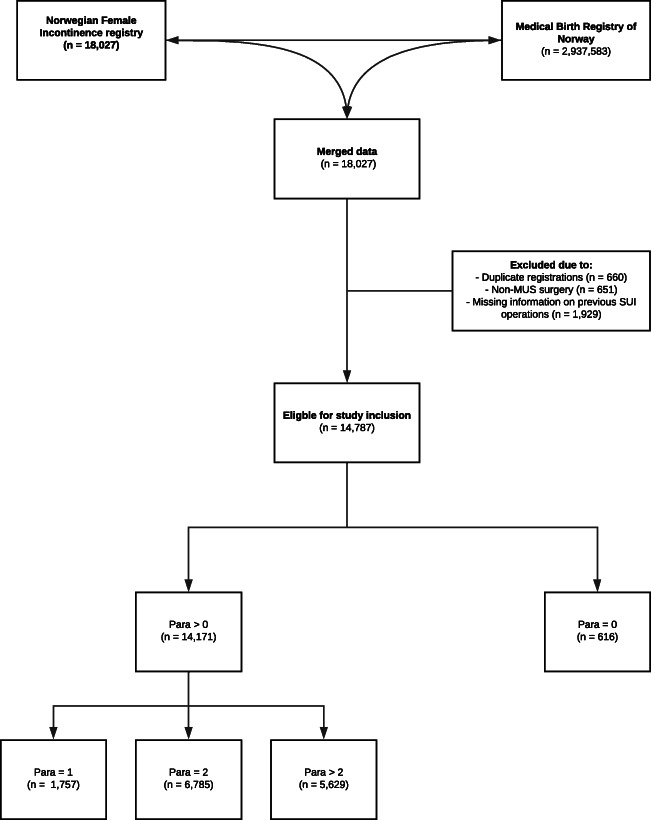
Flowchart

After merging data from the Norwegian Incontinence Registry and the Medical Birth Registry of Norway, 18,027 women from 29 reporting departments who had undergone a mid-urethral sling (MUS) procedure were found eligible for inclusion (Fig. 1). Women with duplicate records, those who had undergone non-MUS surgery for their stress urinary incontinence (SUI) or those who lacked information regarding this being their primary operation or an operation for recurrent SUI were excluded. The remaining 14,787 women were included in the study analyses, of which 14,171 (96%) had undergone at least one childbirth and 616 (4%) were nulliparous before their primary MUS procedure (Fig. 1).

Baseline clinical characteristics and short-term (6–12-month) subjective and objective failure rates for both parous and nulliparous women are presented in Table 1. The median age was 47 years (IQR: 42–52) and median BMI 25.4 kg/m² (IQR: 23.0–29.0) for the total study group. For parous women, the median time from last delivery until MUS operation was 14 years (IQR: 0–46). Additional obstetrical descriptive factors such as parity, age at first delivery, presentation, maximum birth weight, episiotomy, perineal tear grade 3/4, and mode of delivery are presented in Table 1.

**Table 1:** Baseline clinical characteristics, type of surgery, and failure rates (subjective and objective) 6–12 months after MUS for the study groups

Characteristic	All participants (*n* = 14,787)	Para > 0 (*n* = 14,171)	Para = 0 (*n* = 616)	*p*^a^
Age at primary MUS (years)	47 (42–52)	47 (42–52)	50 (44–54)	**< 0.001**
BMI at primary MUS (kg/m^2^)	25.4 (23.0–29.0)	25.4 (23.0–28.7)	27.1 (24.0–30.8)	**< 0.001**
Preoperative stress urinary incontinence index score^b^	8 (7–10)	8 (7–10)	9 (7–10)	**< 0.001**
Preoperative urgency urinary incontinence index score^c^	3 (2–5)	3 (2–5)	4 (2–6)	**< 0.001**
Preoperative cough-jump stress test (g)	33 (0–400)	33 (0–400)	33 (0–300)	0.079
Preoperative maximum urinary flow rate (ml/s)	29 (4–50)	29 (4–50)	27 (6–50)	0.064
Preoperative post-void residual urine (ml)	2 (0–483)	2 (0–483)	1 (0–230)	0.216
Preoperative MUCP^d^ < 20 cmH_2_O	1.8% (107/5808)	1.8% (99/5546)	3.1% (8/262)	0.151
Type of mid-urethral sling				
Retropubic	78.4% (11,599/14,787)	78.5% (11,126/14,171)	76.8% (473/616)	0.317
Inside-out obturator	13.5% (1999/14,787)	13.4% (1898/14,171)	14,9% (92/616)	0.277
Outside-in obturator	4.4% (652/14,787)	4.4% (628/14,171)	3.9% (24/616)	0.609
Mini-sling	3.7% (546/14,787)	3.7 (519/14,171)	4.4 (27/616)	0.330
Objective failure rate^e^	7.7% (832/10,843)	7.6% (786/10,398)	10.3% (46/445)	**0.036**
Subjective failure rate^f^	28.9% (3813/13,205)	28.7% (3630/12,664)	33.8% (183/541)	**0.010**
Time from last delivery until MUS (years)		14 (0–46)		
Parity		2 (1–9)		
Age at first delivery (years)		24 (14–44)		
Maximum birth weight (g)		3820 (770–5680)		
Normal cephalic presentation		88.7% (12,211/13,767)		
Breech presentation		6.9% (949/13,767)		
Abnormal cephalic presentation		4.4% (607/13,767)		
Episiotomy		8.6% (1188/13,767)		
Instrumental vaginal delivery		15.3% (2108/13,767)		
Perineal tear grade 3 or 4		5.4% (746/13,767)		
Cesarean delivery only		2.9% (404/14,171)		

Data are median (25th–75th interquartile range) or % (number/total) unless otherwise specified

Mann-Whitney U test was used when analyzing continuous data; Fisher exact test was used for categorical variables

Entries presented in bold indicates statistically significance (*p* < 0.05)

*MUS* mid-urethral slings, *BMI* body mass index

^a^Para > 0 vs. Para = 0

^b^Stress urinary incontinence index score is calculated from the validated NFIR questionnaire with range 0–12

^c^Urgency urinary incontinence index score is calculated from the validated NFIR questionnaire with range 0–8

^d^Maximum urethra closure pressure

^e^Objective failure defined as 0-g leakage on a cough/jump pad weighing test

^f^Subjective failure defined as stress urinary incontinence index score = 0

Retropubic MUS was the most common sling type utilized (78.4%). For the total population, the subjective and objective 6–12-month failure rate was 28.9% and 7.7%, respectively (Table 1).

In the non-parametric analysis, nulliparous women had a significantly higher subjective and objective short-term failure rate compared with parous woman (subjective failure in 33.8% vs. 28.7%, *p* = 0.010 and objective failure in: 10.3% vs. 7.6%, *p* = 0.036) (Table 1). However, at baseline, the nulliparous women were significantly older (*p* < 0.001), had a higher BMI (*p* < 0.001), and had a higher preoperative symptom load for both stress and urgency urinary incontinence reflected by their higher preoperative stress and urgency urinary incontinence symptom scores (*p* < 0.001) (Table 1).

In the univariate analysis, being nulliparous also seemed to impact both subjective and objective failure rates [OR 1.27 (95% CI 1.05–1.53), *p* = 0.012, and 1.41 (95% CI 1.02–1.95), *p* = 0.037] (Tables 2 and 3). Additional obstetrical factors seemingly impacting failure rates in the univariate analyses were head circumference (subjective failure), age at first delivery (objective failure), and time from last delivery until surgery (objective failure) (Tables 2 and 3). In the multivariate analyses, however, the only obstetrical factor significantly impacting the short-term objective failure rate was being nulliparous before MUS surgery, with an increased risk of 60% [aOR 1.60 (95% CI 1.07–2.40), *p* = 0.022] when compared with parous women having a median of two deliveries (Table 3). For subjective failure, none of the obstetrical factors investigated exerted any significant impact (Table 2). Constitutional factors that were significantly associated with the subjective and/or objective failure rate in the adjusted regression analyses included BMI at time of surgery, preoperative cough-jump stress test results, preoperative stress and urgency symptom loads expressed as preoperative stress and urgency urinary incontinence index scores, preoperative low maximum urinary flow rate, type of sling utilized, and surgical complications (Tables 2 and 3).

**Table 2:** Obstetrical and constitutional risk factors for subjective failure 6–12 months after MUS for the study group (*n* = 14,787)

Obstetric and constitutional variables	Subjective failure % (*N*)^a^	Crude OR (95% CI)	*p*	Adjusted OR (95% CI)	*p*
Parity					
0	33.8 (183/541)	1.27 (1.05–1.53)	**0.012**	1.18 (0.94–1.47)	^*^0.148
1	29.9 (472/1579)	1.06 (0.94–1.20)	0.350	0.95 (0.81–1.12)	0.550
2	28.7 (1742/6074)	Reference		Reference	
≥ 3	28.2 (1415/5011)	0.98 (0.90–1.06)	0.595	0.98 (0.88–1.09)	0.697
Vaginal delivery					
Cesarean delivery only	30.4 (123/404)	1.09 (0.87–1.37)	0.470		
Spontaneous	28.4 (3056/10,762)	Reference			
Instrumental	30.2 (574/1902)	1.09 (0.98–1.21)	0.113		
Presentation					
Normal cephalic	28.5 (3198/11,218)	Reference			
Abnormal cephalic	31.2 (177/597)	1.14 (0.95–1.37)	0.164		
Breech	29.0 (255/879)	1.03 (0.89–1.19)	0.751		
Episiotomy					
No	28.4 (3320/11,597)	Reference			
Yes	30.2 (310/1067)	1.02 (0.89–1.17)	0.769		
Birth weight (g)					
< 4000	29.2 (2374/8119)	Reference			
≥ 4000	27.6 (1255/4544)	0.92 (0.85–1.01)	0.053		
Head circumference (cm)					
< 36	29.8 (1302/4367)	Reference		Reference	
≥ 36	27.8 (2059/7410)	0.91 (0.83–0.98)	**0.019**	0.95 (0.86–1.05)	0.292
Perineal tear 3 or 4					
No	28.7 (3438/11,993)	Reference			
Yes	28.6 (192/671)	1.00 (0.84–1.19)	0.977		
Age at first delivery (years)					
< 25	28.9 (1884/6525)	Reference			
≥ 25	28.4 (1746/6139)	0.98 (0.91–1.06)	0.591		
Age at surgery (years)					
< 30	40.1 (27/66)	1.76 (1.07–2.89)	**0.025**	1.60 (0.87–2.96)	0.129
30–39	29.0 (583/2008)	1.04 (0.93–1.16)	0.494	1.09 (0.95–1.24)	0.231
40–49	28.2 (1796/6359)	Reference		Reference	
50–59	29.4 (1211/4125)	1.06 (0.97–1.15)	0.218	1.01(0.90–1.13)	0.909
60–67	30.2 (195/646)	1.10 (0.92–1.31)	0.297	1.12 (0.86–1.44)	0.402
BMI at surgery (kg/m^2^)					
< 25 *(normal)*	24.5 (1141/4666)	Reference		Reference	
25–29.9 *(overweight)*	29.9 (1066/3570)	1.32 (1.19–1.45)	**< 0.001**	1.25 (1.12–1.39)	**< 0.001**
≥ 30 *(obese)*	36.1 (706/1958)	1.74 (1.56–1.95)	**< 0.001**	1.54 (1.36–1.75)	**< 0.001**
Time from last delivery until surgery (years)					
0–9	27.9 (1081/3875)	0.93 (0.85–1.02)	0.156		
10–19	29.3 (1391/4750)	Reference			
20–29	28.6 (831/2902)	0.97 (0.88–1.07)	0.544		
≥ 30	28.9 (321/1109)	0.98 (0.85–1.13)	0.823		
Preoperative stress urinary incontinence index score^b^					
≤ 6 *(< 25th percentile)*	22.1 (529/2392)	Reference		Reference	
7–10 *(25th–75th percentile)*	29.2 (2502/8583)	1.45 (1.30–1.61)	**< 0.001**	1.22 (1.07–1.38)	**0.003**
11–12 *(> 75th percentile)*	37.4 (553/1478)	2.11 (1.82–2.43)	**< 0.001**	1.42 (1.17–1.72)	**< 0.001**
Preoperative cough-jump stress test (g)					
≤ 13 *(< 25th percentile)*	28.0 (898/3207)	Reference		Reference	
14–67 *(25th–75th percentile)*	28.2 (1892/6704)	1.01 (0.92–1.11)	0.819	0.98 (0.87–1.11)	0.786
68–482 *(> 75th percentile)*	31.0 (1016/3281)	1.15 (1.04–1.28)	**0.009**	1.04 (0.91–1.71)	0.528
Preoperative urgency urinary incontinence index score^c^					
0–1 *(< 25th percentile)*	21.8 (1192//5476)	Reference		Reference	
2–5 *(25th–75th percentile)*	30.1 (1217/4049)	1.62 (1.46–1.78)	**< 0.001**	1.57 (1.39–1.78)	**< 0.001**
6–8 *(> 75th percentile)*	38.9 (1349/3469)	2.99 (2.65–3.39)	**< 0.001**	2.48 (2.10–2.93)	**< 0.001**
Preoperative post-void residual urine (ml)					
< 100	28.9 (3759/13,028)	Reference			
> 100	31.2 (49/157)	1.12 (0.80–1.57)	0.517		
Preoperative maximum urinary flow rate (ml/s)					
≥ 15	28.1 (2982/10,626)	Reference			
< 15	31.5 (123/391)	1.18 (0.95–1.46)	0.143		
Type of mid-urethral sling					
Retropubic	28.0 (2915/10,431)	Reference			
Inside-out obturator	32.4 (556/1717)	1.23 (1.11–1.38)	**< 0.001**	1.18 (1.03–1.35)	**0.021**
Outside-in obturator	30.2 (171/566)	1.12 (0.93–1.34)	0.243	1.26 (1.01–1.58)	**0.043**
Mini-sling	34.8 (171/491)	1.38 (1.14–1.67)	**< 0.001**	1.58 (1.24–2.01)	**< 0.001**
Surgical complications					
No	28.2 (3163/11,2236)	Reference			
Yes	33.0 (650/1969)	1.26 (1.14–1.39)	**< 0.001**	1.26 (1.10–1.43)	**< 0.001**

Data are n (%) unless otherwise specified

Entries presented in bold indicates statistically significance (*p* < 0.05)

*OR* odds ratio, *CI* confidence interval, *MUS* mid-urethral sling, *BMI* body mass index

^a^Postoperative stress urinary incontinence index score > 0

^b^Stress urinary index score is calculated from the validated NFIR questionnaire with range 0–12

^c^Urgency urinary index score is calculated from the validated NFIR questionnaire with range 0–8

^*^Not adjusted for obstetrical factors

**Table 3:** Obstetrical and constitutional risk factors for objective failure 6–12 months after MUS for the study group (*n* = 14,787)

Obstetric and constitutional variables	Objective failure % (*N*)^a^	Crude OR (95% CI)	*p*	Adjusted OR (95% CI)	*p*
Parity					
0	10.3 (46/445)	1.41 (1.02–1.95)	**0.037**	1.60 (1.07–2.40)	^*^0.022
1	7.8 (102/1305)	1.03 (0.83–1.30)	0.754	1.11 (0.81–1.53)	0.568
2	7.6 (380/5028)	Reference		Reference	
≥3	7.5 (304/4065)	0.99 (0.85–1.16)	0.887	0.93 (0.74–1.18)	0.568
Vaginal delivery					
Cesarean delivery only	7.8 (23/295)	1.04 (0.67–1.59)	0.876		
Spontaneous	7.7 (680/8809)	Reference			
Instrumental	6.7 (106/1589)	0.85 (0.69–1.06)	0.146		
Presentation					
Normal cephalic	7.6 (696/9203)	Reference			
Abnormal cephalic	7.1 (32/450)	0.94 (0.65–1.35)	0.723		
Breech	7.8 (58/745)	1.03 (0.78–1.36)	0.825		
Episiotomy					
No	7.6 (722/9542)	Reference			
Yes	7.5 (64/856)	0.99 (0.76–1.29)	0.924		
Birth weight (g)					
< 4000	7.7 (511/6651)	Reference			
≥ 4000	7.3 (275/3746)	0.95 (0.82–1.11)	0.527		
Head circumference (cm)					
< 36	7.4 (268/3624)	Reference			
≥ 36	7.2 (435/6037)	0.97 (0.83–1.14)	0.728		
Perineal tear 3 or 4					
No	7.6 (743/9841)	Reference			
Yes	7.7 (43/557)	1.02 (0.74–1.41)	0.883		
Age at first delivery (years)					
< 25	8.3 (436/5239)	Reference		Reference	
≥ 25	6.7 (350/5159)	0.80 (0.69–0.93)	**0.003**	0.97 (0.76–1.25)	0.819
Age at surgery (years)					
< 30	10.2 (6/59)	1.48 (0.63–3.46)	0.370	1.72 (0.58–5.1)	0.328
30–39	7.8 (132/1696)	1.10 (0.90–1.35)	0.364	1.03 (0.74–1.43)	0.866
40–49	7.1 (369/5181)	Reference		Reference	
50–59	8.4 (288/3414)	1.20 (1.02–1.41)	**0.025**	1.02 (0.74–1.40)	0.898
60–67	7.3 (36/492)	1.03 (0.72–1.47)	0.873	0.73 (0.40–1.35)	0.305
BMI at surgery (kg/m2)					
< 25 *(normal)*	5.7 (214/3781)	Reference		Reference	
25–29.9 *(overweight)*	7.1 (205/2882)	1.28 (1.05–1.56)	**0.016**	1.21 (0.95–1.53)	0.114
≥ 30 *(obese)*	9.8 (154/1575)	1.81 (1.46–2.24)	**< 0.001**	1.48 (1.13–1.93)	**0.004**
Time from last delivery until surgery (years)					
0–9	7.3 (235/3241)	1.06 (0.89–1.27)	0.519	1.22 (0.90–1.65)	0.207
10–19	6.9 (267/3893)	Reference		Reference	
20–29	8.5 (201/2372)	1.26 (1.04–1.52)	**0.019**	1.00 (0.71–1.39)	0.981
≥ 30	9.5 (83/870)	1.43 (1.11–1.85)	**0.006**	1.61 (0.99–2.62)	0.056
Preoperative stress urinary incontinence index score^b^					
≤ 6 *(< 25 percentile)*	6.6 (127/1921)	Reference		Reference	
7–10 *(25th–75th percentile)*	7.4 (524/7111)	1.12 (0.92–1.37)	0.255	1.02 (0.77–1.36)	0.880
11–12 *(> 75th percentile)*	10.6 (129/1220)	1.67 (1.29–2.16)	**< 0.001**	1.04 (0.70–1.55)	0.856
Preoperative cough-jump stress test (g)					
≤ 13 *(< 25th percentile)*	5.7 (141/2488)	Reference		Reference	
14–67 *(25th–75th percentile)*	7.1 (393/5513)	1.28 (1.05–1.56)	0.016	1.30 (0.96–1.74)	0.095
68–482 *(> 75th percentile)*	10.4 (296/2837)	1.94 (1.58–2.39)	**< 0.001**	1.96 (1.43–2.68)	**< 0.001**
Preoperative urgency urinary incontinence index score^c^					
0–1 *(< 25th percentile)*	6.1 (158/2612)	Reference		Reference	
2–5 *(25th–75th percentile)*	7.2 (464/6437)	1.21 (1.00–1.45)	0.049	1.19 (0.91–1.57)	0.197
6–8 *(> 75th percentile)*	11.9 (194/1637)	2.09 (1.68–2.60)	**< 0.001**	1.89 (1.34–2.67)	**< 0.001**
Preoperative post-void residual urine (ml)					
< 100	7.6 (815/10,693)	Reference			
> 100	10.4 (14/135)	1.40 (0.80–2.45)	0.235		
Preoperative maximum urinary flow rate (ml/s)					
≥ 15	6.5 (563/8719)	Reference			
< 15	10.4 (33/317)	1.68 (1.16–2.44)	**0.006**	1.67 (1.09–2.57)	**0.018**
Type of mid-urethral sling					
Retropubic	6.8 (578/8539)	Reference			
Inside-out obturator	10.4 (151/1446)	1.61 (1.33–1.94)	**< 0.001**	1.90 (1.47–2.4)	**< 0.001**
Outside-in obturator	13.5 (59/436)	2.16 (1.62–2.87)	**< 0.001**	2.71 (1.87–3.92)	**< 0.001**
Mini-sling	10.4 (44/422)	1.60 (1.16–2.12)	**0.004**	1.63 (1.02–2.58)	**0.040**
Surgical complications					
No	7.5 (687/9219)	Reference			
Yes	8.9 (144/1623)	1.21 (1.00–1.46)	**0.048**	1.36 (1.03–1.80)	**0.028**

Data are *n* (%) unless otherwise specified

Entries presented in bold indicates statistically significance (*p* < 0.05)

*OR* odds ratio, *CI* confidence interval, *MUS* mid-urethral sling, *BMI* body mass index

^a^Postoperative cough-jump pad-weighing test = 0 g

^b^Stress urinary incontinence index score is calculated from the validated NFIR questionnaire with range 0–12

^c^Urgency urinary incontinence index score is calculated from the validated NFIR questionnaire with range 0–8

^*^Not adjusted for obstetrical factors

## Discussion

This nationwide prospective cohort study based on high-quality registry data investigated the potential impact of women’s obstetrical history expressed as various obstetrical factors on short-term subjective and objective failure rates after MUS surgery. The results initially indicated that several obstetrical variables might exert an impact on both outcomes as shown in the univariate analyses. However, in the final multivariate model when adjusting for known constitutional factors, none of the obstetrical variables remained independent risk factors for subjective failure. Only nulliparity before MUS surgery remained an independent risk factor for objective failure. Risk factors such as high BMI at time of surgery, a high preoperative degree of objective leakage, a high preoperative symptom load for both stress and urgency symptoms, preoperative maximum urinary flow rate < 15 ml/s, type of sling utilized and surgical complications, however, remained strong risk factors for both subjective and objective outcomes in the multivariate model. These constitutional risk factors for SUI have previously been well documented in the literature [11, 16, 21, 22].

Childbearing is identified as an important independent risk factor for SUI. In a large cross-sectional study including 27,900 women in Norway, SUI prevalence was associated with increasing parity, with a relative risk of 1.9 (95% CI, 1.6–2.2) for primiparous women and 2.3 (95% CI, 2.0–2.6) for women with two deliveries [6]. The association between childbearing and urinary incontinence is, however, not fully understood. Data suggest that pregnancy and delivery induce pelvic floor injury through mechanical forces [8], causing increased bladder neck hypermobility and decreased pelvic floor contractility [23, 24]. Parous women have an attributable risk of 50% for SUI development related to pregnancy and childbirth [9]. Furthermore, several studies have shown that vaginal delivery is a significant risk factor for the development of SUI [4, 5, 9, 25].

However, to date it is unknown whether these obstetrical factors such as delivery mode, obstetric anal sphincter injury, levator ani avulsion, and fetal factors also impact surgical outcomes in women with SUI. Parity is, to our knowledge, the only obstetrical factor previously studied [13, 15]. Laterza et al. evaluated the effect of parity at the time of surgery on short- and long-term outcomes in women who underwent SUI surgery by MUS [15]. This study utilized data from a previously published randomized controlled trial that included 554 patients in which subjective and objective outcomes of TVT and TVT-O were compared. They found no significant association between parity and the risk of either subjective or objective SUI recurrence. Furthermore, in a prospective cohort study by Majkusiak and co-workers including 238 women who had a retropubic MUS operation, the number of vaginal deliveries showed no impact on short-term sling failure [13]. Thus, the findings of both of these studies are consistent with the results in the present study. However, as we had the possibility of also including a sufficiently large group of women who had not given birth before their MUS operation, we were able to demonstrate that being nulliparous before MUS surgery seems to be an independent risk factor for objective failure compared with parous women, even after adjusting for obstetrical and known constitutional risk factors. We know from published literature that even though childbirth is a risk factor for stress urinary incontinence, studies have also demonstrated that stress urinary incontinence occurs in approximately 5%–11% of nulliparous women 25–64 years old [6, 26]. It is reasonable, therefore, to hypothesize that nulliparous women with SUI might have a different or additional underlying pathophysiological mechanism for their incontinence rather than a simple urethral hypermobility caused by trauma to the pelvic floor. In the present study, the nulliparous women had a higher BMI, which by itself is a known risk factor for SUI [16]. There is also evidence that hereditary factors could play a part, such as a different connective tissue composition in nulliparous women with increased risk of developing SUI. This was demonstrated in a study from Keane et al. which found significantly less periurethral collagen in nulliparous women with pure SUI compared to continent nulliparous controls, indicating a genetically higher susceptibility to SUI development [27]. Other potential explanations for SUI developing in nulliparous women with no known trauma to the pelvic floor could include a poor sphincter function of unknown cause, a dysfunctional pelvic floor due to age-related atrophy of muscles, and/or undiagnosed neurological diseases. It has been demonstrated that women with sphincter insufficiency have lower cure rates after MUS compared to women with urethral hypermobility [12].

Constitutional factors were confirmed in this study as strong risk factors for both subjective and objective short-term failure. In population-based studies high BMI has proved to be one of the main risk factors for both the development of stress urinary incontinence [28] and poor outcomes after MUS [16]. Retropubic MUS are considered to be more effective than trans-obturator and mini-slings [11]. This was also confirmed in our study demonstrating a significantly lower failure rate when retropubic MUS was utilized. A high preoperative urgency symptom load almost doubled the odds of subjective and objective failure after MUS surgery in our study, which is in agreement with previous published studies [21, 22]. Several studies have also found advancing age to be a risk factor for MUS failure [14, 21, 29]. In our study, age at surgery was not a risk factor for subjective or objective failure. However, we believe the reason for this are that women > 67 years (born before 1949) were excluded from our study as these could theoretically have given birth to non-registered children before the inception of the MBRN. In a study by Engen et al., also based on data from approximately the same cohort of women, it was demonstrated that a significant increase in MUS failure only occurred for women in the 7th decade and older [29].

This study’s main strength was the use of data from a large population-based cohort. The cohort was identified through a surgical incontinence registry with proven high coverage and data reliability [18]. All inhabitants in Norway are provided with a public healthcare system where surgical treatment for urinary incontinence is free of charge. This ensured a low degree of selection bias and a high degree of statistical precision in the analysis. Consequently, we believe the present study has a high level of external validity. Furthermore, we were also able to explore the women’s obstetrical history in detail because of the merging with data from the Medical Birth Registry of Norway (MBRN), which has been proven suitable for population-based studies [30].

There are also several important limitations to this study, the most important being that we were only able to study the potential impact of obstetrical factors on short-term results, as the NFIR only has high coverage for the first postoperative 6–12 months of follow-up. Later follow-ups are optional for the reporting hospitals and were therefore deemed to have a too high risk of selection bias to be used in the present study. We realize that a potential impact from obstetrical factors on long-term results therefore cannot be ruled out with certainty based on our study. Other important limitations to this study were the inability to adjust for factors related to the surgeon’s experience (surgical volume), local differences in incontinence assessment, and local preferences regarding the type of sling. Furthermore, all use of registry data carries the risk of missing data and inaccuracy in the individual entries, potentially impacting the results, and should always be interpreted with that in mind.

Knowledge about factors associated with treatment failure is essential when counseling patients regarding MUS surgery’s efficacy and to facilitate appropriate expectations of women with SUI women prior to their decision on surgery. Although pregnancy and childbirth are considered significant risk factors for developing stress urinary incontinence, these obstetrical factors do not seem to majorly impact the short-term surgical results. However, the negative impact on objective failure rates of being nulliparous before MUS surgery calls for further research to fully understand the mechanism behind the development of SUI in this sub-group of women.

## Supplementary Information

ESM 1 (PDF 1135 kb)
