# Supplementary material for: Previous obstetrical history does not impact short-term mid-urethral sling outcomes
Source: Int Urogynecol J. 2021 May 14;32(7):1733–43. doi: 10.1007/s00192-021-04836-5 (PMC8295164; doi:10.1007/s00192-021-04836-5)
Supplement: Supplementary file 1 — (PDF 1135 kb) [file 192_2021_4836_MOESM1_ESM.pdf]

Norwegian female incontinece questionnaire for urinary incontinence

Pre and postoperative recording

Date for questionnaire completion ☐☐.☐☐.☐☐ patient number ☐☐☐☐

Please answer all questions.

(Mark yes, no or not relevant for each alternative in question 1)

1. Do you leak urine?

	yes	no	not relevant
when you cough	☐	☐	☐
when you sneeze	☐	☐	☐
when you laugh	☐	☐	☐
when you walk up or down in stairs	☐	☐	☐
when you raise from bed	☐	☐	☐
hen you lift heavy objects	☐	☐	☐
during physical activity (running to catch the bus)	☐	☐	☐
during sports	☐	☐	☐
during intercourse	☐	☐	☐

(Mark only one alernative for each question from 2 - 6))

2. **How often do you leak urine in relation to physical activity, when you laugh, cough or sneeze?**

- ☐ never
- ☐ 1 -4 times each month
- ☐ 1 -6 times each week
- ☐ once per day
- ☐ more than once per day

3. **How large is the amount of urine you usually leak during physical activity or when you laugh, cough or sneeze?**

- ☐ nothing
- ☐ drops / moist underwear
- ☐ dripping / wet underwear
- ☐ running / passes through all your cothes
- ☐ running down your legs or down at the floor

4. **How often do you experience sudden and imperious need to void and urinary lekage before you reach the toilet?**

- ☐ never
- ☐ 1 -4 times each month
- ☐ 1 -6 times each week
- ☐ once per day
- ☐ more than once per day

5. **How large is the amount of urine you usually leak when you experience sudden and imperious need to void and**

- ☐ nothing
- ☐ drops / moist underwear
- ☐ dripping / wet underwear
- ☐ running / passes through all your cothes
- ☐ running down your legs or down at the floor

6. **If you experience symptoms both as those described in question 2 and question 4, what is troubling you the most?**

- ☐ leakage during physical activities more than leakage related to urge?
- ☐ leakage during urgency more than during physical activity?
- ☐ equally trouble by leakage during urgency as by lekage during physical activity?
- ☐ I don't have leakage as described in question 2 nor question 4

(Mark only one alternative for each question 7 - 11)

7. **How many incontinence pads do you use?**

☐ none ☐ 1 - 3 per week ☐ 4 - 6 per week ☐ 1 - 4 per day ☐ more than 4 per day

8. **How many times have you been treated for cystitis the last 6 months?**

☐ none ☐ once ☐ 2 - 3 times ☐ 4 times ☐ more than 4 times

Draft

Norwegian female incontinece questionnaire for urinary incontinence

9. How often do you avoid activities (for example a hobby, physical training or going out) because you are afraid of leaking urine)?

☐ never ☐ seldom ☐ sometimes ☐ often ☐ always

10. How often do you avoid places and situations where you are awarre of that a toilet is not easily avalible?

☐ never ☐ seldom ☐ sometimes ☐ often ☐ always

11. Is your saxual life influenced by your leakage problem? (To be answered before treatment)

☐ not relevant
☐ substantially improved ☐ some improvement ☐ unchanged ☐ some deterioration ☐ substantial deterioration

(Mark yes, no or not relevant for each question under 12)

12. Does your urinary leakage influence?	yes	no	not relevant
your vacation?	☐	☐	☐
your family life?	☐	☐	☐
your social life (going out, meeting friends?)	☐	☐	☐
your sleep?	☐	☐	☐

(Mark only one alternative for each question 13 - 14)

13. Is your sexual life influenced by your leakage problem? (To be answered after treatment)

☐ not relevant
☐ substantially improvement ☐ some improvement ☐ unchanged ☐ some deterioration ☐ substantial deterioration

14. Are you satisfied with the result of the treatment you have received to cure your urinary leakage?

☐ very satisfied ☐ moderately satisfied ☐ neither satisfied nor unsatisfied ☐ moderately unsatisfied ☐ very unsatisfied

Please do not fill in. Will be filled in by a physician

☐☐ investigator ID number

date of incontinence surgery

☐☐ ☐☐ ☐☐

☐☐ surgeon ID number

☐☐ surgical procedure number

☐ number of earlier surgical procedures to cure incontinence

☐ yes ☐ no incontinence surgery combined with vaginal surgery

☐ yes ☐ no incontinence surgery performed previously in our department

☐☐ number of micturitions 24 hours

☐☐☐ mean voiding volume ml.

☐☐☐ leakage during 24 hours pad test gr.

maximum closure pressure cm H2O ☐☐

leakage during stress test gr. ☐☐☐

residual urine ml. ☐☐☐

maximum flow ml/sec. ☐☐

height in cm. ☐☐☐

weight in kilo ☐☐☐

date of stopped and not completed incontinence surgery

☐☐ ☐☐ ☐☐

date of tape surgeically adjusted

☐☐ ☐☐ ☐☐

date of tape transected

☐☐ ☐☐ ☐☐

complication ☐ yes ☐ no
bladder perforation ☐ yes ☐ no
hematoma > 3 cm ☐ yes ☐ no
superficial vound infection ☐ yes ☐ no
deep vound infection ☐ yes ☐ no
catheter > 1 week ☐ yes ☐ no
catheter > 1 month ☐ yes ☐ no
tape adjusted surgically ☐ yes ☐ no
gut perforation ☐ yes ☐ no
vascular lesion ☐ yes ☐ no
bleeding > 500 ml. ☐ yes ☐ no
urethral lesion ☐ yes ☐ no

pain 0 to 10 ☐

duration of pain
☐ none
☐ less than 2 weeks
☐ 2 to 12 weeks
☐ more than 12 weeks

other complications

☐☐☐☐☐☐☐☐☐☐☐☐☐☐☐☐☐☐☐☐☐☐☐☐☐☐☐

☐☐☐☐☐☐☐☐☐☐☐☐☐☐☐☐☐☐☐☐☐☐☐☐☐☐☐

Draft
